# Wearable Technology for Detecting Significant Moments in Individuals with Dementia

**DOI:** 10.1155/2019/6515813

**Published:** 2019-09-25

**Authors:** Chelsey Lai Kwan, Yacine Mahdid, Rossio Motta Ochoa, Keven Lee, Melissa Park, Stefanie Blain-Moraes

**Affiliations:** ^1^Biosignal Interaction and Personhood Technology Lab, McGill University, Montreal, Quebec H3G 1A4, Canada; ^2^School of Physical and Occupational Therapy, McGill University, Montreal, Quebec H3G 1Y5, Canada

## Abstract

The detection of significant moments can support the care of individuals with dementia by making visible what is most meaningful to them and maintaining a sense of interpersonal connection. We present a novel intelligent assistive technology (IAT) for the detection of significant moments based on patterns of physiological signal changes in individuals with dementia and their caregivers. The parameters of the IAT are tailored to each individual's idiosyncratic physiological response patterns through an iterative process of incorporating subjective feedback on videos extracted from candidate significant moments identified through the IAT algorithm. The IAT was tested on three dyads (individual with dementia and their primary caregiver) during an eight-week movement program. Upon completion of the program, the IAT identified distinct, personal characteristics of physiological responsiveness in each participant. Tailored algorithms could detect moments of significance experienced by either member of the dyad with an agreement with subjective reports of 70%. These moments were constituted by both physical and emotional significances (e.g., experiences of pain or anxiety) and interpersonal significance (e.g., moments of heighted connection). We provide a freely available MATLAB toolbox with the IAT software in hopes that the assistive technology community can benefit from and contribute to these tools for understanding the subjective experiences of individuals with dementia.

## 1. Introduction

The prevalence of dementia is expected to dramatically increase in the coming decades: in Canada, it is anticipated that the number of individuals over the age of 65 living with dementia will double within the next 20 years [1]. Dementia is a set of symptoms caused by disorders and diseases affecting the brain, including Alzheimer's disease and vascular dementia. Individuals with dementia typically experience memory loss, difficulties with problem-solving, language and orientation, and altered mood and behavior. These symptoms diminish the individual's ability to perform activities of daily living and disrupt their relationships, often reducing their ability to participate in society and requiring significant support in their daily lives. At present, hospitals in Canada already provide full-time care to approximately 51,000 individuals with dementia [1], and almost half a million more are cared for by family and informal caregivers. The indirect annual cost of caring for those with dementia is conservatively estimated at $1.2 billion; this estimate is projected to double by 2031 [1].

In response to the escalating demands of caring for persons with dementia, intelligent assistive technologies (IATs) for this population have proliferated. IATs have been developed for nearly every aspect of daily living and range from distributed systems (e.g., smart homes and integrated sensor systems), personal robots (e.g., socially/physically assistive), mobility and rehabilitation aids (e.g., powered wheelchairs and electronic canes), handheld/multimedia devices (e.g., smart phones, personal digital assistants (PDAs), and tablets), software applications (mobile or web-based apps), voice-prompting systems [2], wearable devices (e.g., smartwatches and e-textiles), and human-machine interfaces (HMIs) [3]. Of particular note is the growth in wearable technologies for this population, which are primarily used to monitor the well-being and safety of individuals with dementia. Wearables have been employed to track sleep and toileting patterns [4], to aid memory retrieval [5], and to aid way finding and navigation [6–9].

While the majority of IATs for individuals with dementia have focused on maintaining and improving their functional and cognitive abilities, there has been minimal focus on tracking the subjective experience of individuals with dementia. Understanding the subjective emotional and mental experience of an individual with dementia has important implications for the quality of life of these individuals and their caregivers (familial and professional). Insight into the significant events for individuals with dementia can make visible what is meaningful to them [10] and can, thus, empower caregivers to understand and ameliorate care decisions based on what matters to them [11], as well as intervene before a minor annoyance or frustration can build into an outburst or an aggressive behavior. Moreover, the communication of meaning and important moments is critical to maintaining a sense of connection and thus to preserving relationships between individuals with dementia and their caregivers [12]. In this paper, we describe a novel, wearable IAT that can track and detect moments of significance for individuals with dementia. As dementia is associated with progressively declining cognitive and motor abilities, this IAT focuses upon detecting significant moments from their physiological signals.

The feasibility of detecting significant mental and emotional reactions from changes in physiological signals has long been established [13]. Physiological signals have been used to provide insight into an individual's significant reactions in fields such as polygraphy [14] and biofeedback [15] for decades. Additionally, physiological signals such as electrodermal activity (EDA), heart rate, and skin temperature are amenable to being recorded from noninvasive, wearable sensors. The IAT presented in this paper records physiological signals from a device worn on the hand and uses a custom software to detect individual-specific moments of significance from changes in their physiological patterns. We demonstrate that this IAT is able to accurately detect moments of physiological significance and moments experienced as significant interpersonal interactions in individuals with dementia and their caregivers. Furthermore, we make the algorithms and software freely available through a Matlab toolbox, which can be effectively used to detect moments of significance from patterns of physiological signals recorded from wearable sensors.

## 2. Materials and Methods

### 2.1. Description of Assistive Technology

#### 2.1.1. Hardware

Autonomic nervous system (ANS) signal data were collected from a wearable device called the Triple Point Sensor (TPS) (Thought Technology Ltd. ©). The TPS was designed to be worn on the fingertip by securing it with a loose elastic or Velcro strap. Three physiological signals were recorded at a sampling frequency of 15 Hz: (1) electrodermal activity (EDA); (2) skin temperature; and (3) heart rate (HR). Signals were transmitted via Bluetooth to a paired Android phone, which stored the data in a custom database.

#### 2.1.2. Software

Custom software, called *Events Finder*, a freely available MATLAB toolbox (https://github.com/BIAPT/Events-Finder), was designed to detect salient moments from individual-specific physiological signatures. Broadly, the software detected characteristics from the ANS signals that are known a priori to be associated with changes in emotional state. Analysis of the EDA signal focused on the detection of electrodermal reactions (EDRs)—transient increases of 0.05 *µ*s or more within 10 seconds, which have been associated with heightened emotional arousal [16, 17]. Skin temperature analysis focused on detecting changes between vasoconstrictor and vasodilatory responses [18], and the heart rate was monitored for unusual patterns of acceleration and deceleration [19]. The software consists of three components: (a) preprocessing; (b) signal quality assessment; (c) event detection, which are detailed in the sections below.

**Figure 1:**
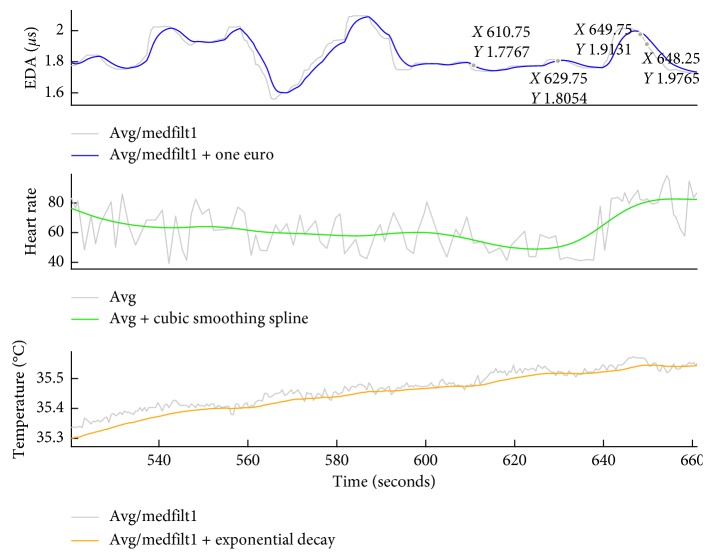
Preprocessing of autonomic nervous system signals. Modality-specific filters were applied to each physiological signal to enhance salient features.

*(1) Preprocessing*. Each of the three ANS signals was preprocessed to remove nonphysiological artifacts. Filters were chosen to be compatible with real-time processing, to ensure that the final software had translational potential for real-world use. First, missing datapoints or datapoints that resulted from hardware error were identified by flagging samples with (1) a value of 0 and (2) whose value was greater or less than three standard deviations from the average of the preceding second of data. These datapoints were replaced by interpolation using a 1-D median filter (EDA filter order *n* = 75; skin temperature filter order *n* = 1). Second, all signals were sent to a moving average filter with a non-overlapping, 0.5-second window; the resulting smoothed data had a sampling frequency of 2 Hz. Third, the signals were sent to a modality-specific filter that enhanced particular features associated with a salient reaction. To minimize jitter and lag in the EDA signal, a *one Euro* filter was applied with parameters mincutoff = 50 and beta = 4 [20]. An exponential decay filter was applied to the skin temperature signal to remove the high-frequency noise inherent in the signal (smoothing parameter *p*=0.95). Finally, the cubic smoothing spline function from MATLAB was applied to the heart rate data (smoothing parameter *p*=0.001). The effects of these modality-specific filters on their respective physiological signals are illustrated in Figure 1.

*(2) Signal Quality Assessment*. After preprocessing, a signal quality index (SQI) algorithm was applied on a sliding window of 0.5 seconds across all three physiological signals. The index ranged from SQI_*x*_ = 1, when data from physiological signal *x* was physiologically valid, to SQI_*x*_ = 0, when data from physiological signal *x* was wholly contaminated by noise resulting from the actions of the user, such as shifting the sensor's position on the hand, scratching around the sensor, and applying pressure to the sensor. EDA signals were assigned a lower SQI_EDA_ when the rate of change exceeded physiological possibility, when it registered no signal for 25 seconds or longer, or when the absolute value exceeded the bounds of a normal physiological range. Skin temperature signals were assigned a lower SQI_temp_ when it registered no signal for 25 seconds or longer, or when values were less than 15 degrees Celsius (e.g., below normal physiological range). The specific parameters used to generate SQI_*x*_ are reported in Table 1. Upon completion of a data recording session, the mean (*µ*) and standard deviation (*σ*) of the SQI_*x*_ for each individual physiological signal were calculated. Subsequently, a binary time series (SQI_all_) of non-overlapping 0.5 second steps was created, where SQI (*t*)_all_ = 0 if SQI (*t*)_EDA_ < *μ* (SQI_EDA_) − *σ* (SQI_EDA_) or SQI (*t*)_temp_ < *μ* (SQI_temp_) − *σ* (SQI_temp_), and SQI (*t*)_all_ = 1 otherwise. Any datapoint where SQI (*t*)_all_ = 0 was not considered in the subsequent event detection algorithm.

*(c) Physiological Event Detection*. The objective of this subcomponent was to detect events in the cleaned, high-quality data that corresponded to a salient moment for the user. The dominant physiological modality that manifests emotional processing varies from individual to individual [21, 22], and the characteristics of the changes within this dominant modality also vary according to multiple factors, including sex, age, and time of day [23]. Thus, in broad terms, event detection required (1) tuning the parameters for detecting specific features within each signal and (2) varying the relative weight of contribution of each of the three physiological signals.

Signal-specific feature detection consisted of a set of adjustable parameters for each physiological modality. Within the EDA signal, electrodermal reactions (EDRs) are associated with an orientation response and heightened emotional arousal [16, 17]. Canonically, an EDR consists of an increase greater or equal to 0.05 *μ*S within a 10-second interval [16]. Tuning the event detector to capture EDRs for each individual required varying (1) *t*_EDR_, defined as the time interval within which to search for an EDR and (2) *A*_EDR_, defined as the minimum amplitude (*μ*s) increase across *t*_EDR_ that was considered to be an EDR. Heart rate has natural patterns of acceleration and deceleration, driven by factors such as respiratory sinus arrhythmia [19, 24]. Salient events were detected at time points where heart rate acceleration and deceleration varied outside of these baseline patterns. Acceleration and deceleration patterns were tracked using the *find_peaks* algorithm in MATLAB. Tuning the event detector to capture significant changes from these patterns required varying *minPeakProminence*, the threshold for which one peak must deviate with respect to height and location from the other peaks in the heart rate time series to be flagged as an event. Fingertip temperature changes associated with salient responses are driven by transient changes in the cutaneous microcirculation [18, 25]. Vasoconstriction and vasodilatory responses manifest as changes between the rate of cooling and heating in the fingertip. The event detector for fingertip temperature was tuned by varying (1) *t*_temp_, defined as the time interval within which to consider trends in temperature and (2) *T*_max_ − *T*_min_, defined as the difference between the maximum and minimum temperature (°C) within the interval *t*_temp_.

Applying the above rules for detecting features in each physiological modality yielded three time series, with a step size of 0.5 seconds, and values corresponding to the strength of each feature extracted from the physiological data: EDR (*t*), Δtemp(*t*), and HR_var_ (*t*). Subsequently, the three physiological modalities were assigned a weighting factor to scale the relative contribution of each signal to the overall score *S*, indicating the magnitude of change in the weighted physiological features.

(1) $$\begin{array}{c} S=a\text{EDR}\left(t\right)+b\Delta \text{temp}\left(t\right)+c{\text{HR}}_{\mathrm{var}}\left(t\right), \end{array}$$

where 0 ≤ *a*, *b*, *c* ≤ 1. All three weighting factors (*a*, *b*, and *c*) where initialized to 1 and adjusted in an iterative fashion as described in Section 2.3.

### 2.2. Study Design

#### 2.2.1. Participants

Participants were recruited from a nonprofit organization that provided a variety of services to the community for individuals with dementia and their caregivers. The assistive technology was calibrated and tested on three dyads, each dyad consisting of an individual with dementia and his/her primary caregiver. Pseudonyms and the relationship between members of the dyad are listed in Table 2. Written consent was obtained from all caregivers for themselves and for the individual with dementia—who also provided written assent—after a careful discussion of risks and benefits. This study was approved by the Institutional Review Board of McGill University (A06-B25-17B).

#### 2.2.2. Data Collection

Participants engaged in an 8-week movement-based program and ran over the course of 15 weeks. The program was held at the nonprofit organization—a familiar environment that was chosen to support the engagement and self-expression of the individuals with dementia [26]. Prior to the beginning of each session, each member of the dyad was outfitted with the assistive technology. Participants were given the choice of securing the sensor onto their fingertip or onto the palm of their hand based on comfort and ease of movement. Sensors were secured with a Velcro band. Collection of ANS data from the sensors was initiated from a paired Android smartphone prior to the movement session by a research assistant. Sessions were also video and audio recorded by using a video camera mounted near the ceiling; audiovisual data were timestamped and synchronized with the ANS data from each participant.

Following each 45-minute movement session, ANS data from each participant were run through the *Events Finder* software described above. Events with the highest *S* score were selected, and the video recording was spliced 10 seconds prior to and 10 seconds following each event, resulting in 10-second–20-second clips of physiologically triggered salient moments for each dyad.

After selected sessions, these 20-second video clips were presented to the respective dyad on a laptop computer. Dyads were interviewed to assess whether or not the salient moments detected by the algorithm corresponded to a subjective recollection of a significant experience. Significant experiences were defined as events [10] or heightened moments that stood out and/or were memorable (e.g., see also [27]). A research assistant guided their reflection about the moments captured in the video using the following questions:

- What was happening at this point during the session? Was this in relation to your partner?
- Did this moment stand out to you from the ordinary flow of the session? If yes/no, why?
- Are there moments we did not go over where you felt an especially strong sense of connection or disconnection to your partner?

Participant responses were audio recorded and used to adjust the parameters of the event detection algorithm.

### 2.3. Personalization of the Assistive Technology

Prior to the first movement session, all parameters of the *Event Finder* algorithm were set to default values. All physiological modalities were equally weighted (i.e., *a* = *b* = *c* = 1). Default parameters for feature detection within each physiological signal were empirically derived from preliminary data: electrodermal reactions were initiated as increased in 0.05 *μ*s across 10 seconds (i.e., *t*_EDR_ = 10; *A*_EDR_ = 0.05); heart rate acceleration and deceleration were flagged at *minPeakProminence* = 10 bpm; skin temperature trends were considered to change if there was a change of 0.01°C across 10 seconds (i.e., *T*_max_ − *T*_min_ = 0.01; *t*_temp_ = 10).

After each of the first four sessions, a research assistant reviewed the physiological data of each participant to individually tailor each of the parameters to match the particular idiosyncratic patterns of his/her physiological responsiveness. First, the audiovisual recording was observed in full for any moments that stood out from the ordinary flow of the session. Time points associated with these moments were flagged on the synchronized physiological signal recording. For each of these time points, a 10-second epoch surrounding each event was segmented, and EDR (*t*), Δtemp(*t*), and HR_var_ (*t*) were calculated. The parameter value (Table 1) associated with each feature was calculated and used to replace the default parameters. Subsequently, the *Event Finder* algorithm was re-run, and the accuracy of detection of significant moments was calculated. The parameter set that produced the highest accuracy was retained for the next movement session.

At the end of some of the last four sessions, the algorithm parameters were iteratively adjusted to align with participants' subjective report of the experience associated with detected physiological responses. Participants were presented with 20-second video clips containing salient moments detected by the personalized *Event Finder* algorithm. Their responses of whether or not the video detected a significant event were used to iterate on the parameters to decrease the overall score *S* of the nonsignificant moments. If participants reported significant moments that were not detected by the algorithm, parameters were adjusted to increase the overall score *S* of these moments. The algorithm with the adjusted parameters was used to detect significant moments in the participant's next movement session.

### 2.4. Data Analysis

For all sessions where participants provided feedback about the salience of the moments identified by *Event Finder*, two research assistants independently reviewed the video and interview data to assess the performance of the assistive technology. True positives (TPs) consisted of events identified by the algorithm that corresponded to a salient moment for the user. These moments were recognized by the participant's ability to vividly recall their subjective experience in the video clip associated with the event. False positives (FPs) consisted of events identified by the algorithm that did not correspond to a salient moment for the user. FPs occurred when (1) the event was flagged as a result of a signal artifact (e.g., the video showed the user adjusting the sensor) or (2) the participant could not recall what happened at the moment depicted in the video, or described only what he/she was observing in the video. All true-positive events were further coded to assess whether they corresponded to a moment of physiological significance (TP_phys_) or a moment of interpersonal significance (TP_pers_). TP_phys_ included vivid recollections of subjective feelings of discomfort, effort, pain, surprise, relaxation, or stress in relation to the event. TP_pers_ were defined as events where participants described positive or negative feelings induced by interactions with another individual. These events were typically accompanied by an underlying explanation or story.

## 3. Results

Each dyad participated in 2-3 sessions where they provided oral feedback that was used to calibrate *Event Finder* and to assess the performance of the algorithm. The total number of events per session presented to the dyads for feedback varied according to Table 3, with fewer events in the earlier sessions to help dyads become accustomed to the interview process. The session ID reflected the state of the internal parameters of the algorithm (e.g., default or customized).

### 3.1. Persons with Dementia Have Distinct, Personal Characteristics of Physiological Responsiveness

**Figure 2:**
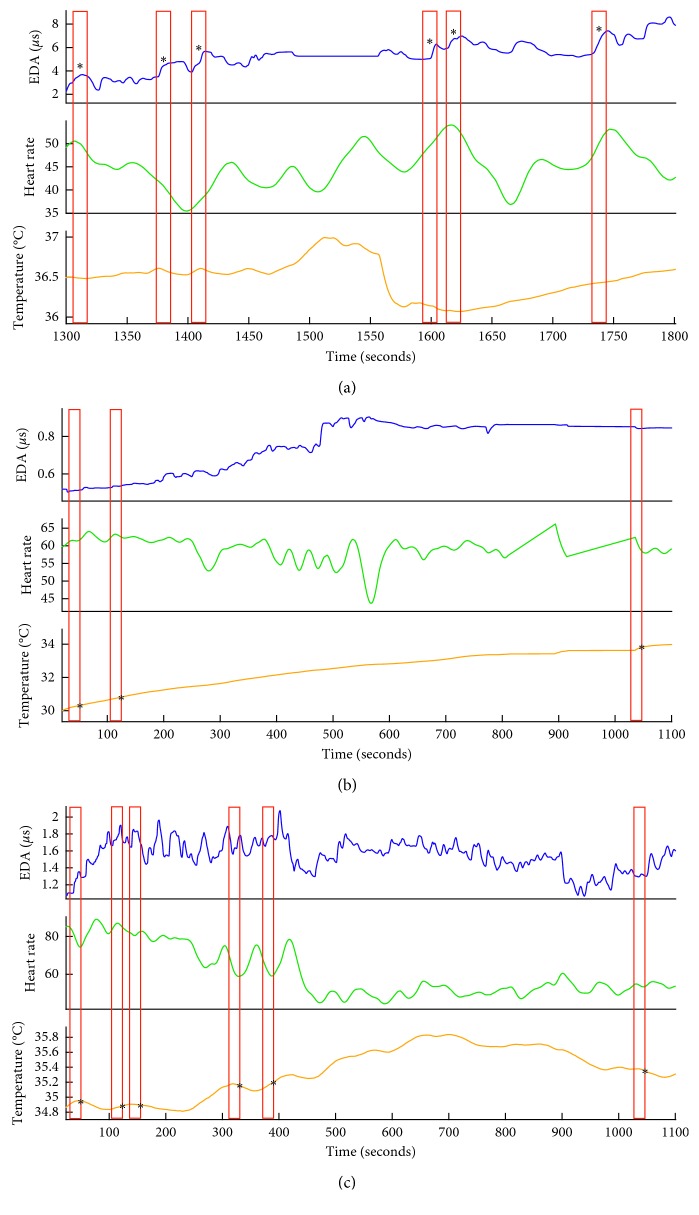
True positive events detected for participants with dementia in their final movement session. Preprocessed and quality-checked signals for electrodermal activity (blue), heart rate (green), and skin temperature (yellow) are presented for Mary (a), Elisa (b), and Irene (c). Red boxes indicate the 30-second “event” that was detected by the final tailored algorithm using parameters presented in Table 4. For each detected event, ^*∗*^ represents the physiological modality dominating each change. Each participant presents varying patterns of physiological responsiveness, and their event-detection algorithm is dominated by different physiological modalities, illustrating the need for personalizing the software for each individual.

While the *Events Finder* software was initialized with the same default parameters across all participants, individual-specific patterns were identified by the end of the fourth session. The tailored parameters used in *Events Finder* at the end of the final session are presented in Table 4 for all participants with dementia. Mary's salient reactions were characterized by EDRs, which morphologically increased sharply in amplitude (i.e., 0.24 *μ*s in 10 seconds) (Figure 2(a)). Elisa's salient reactions were manifest in heart rate accelerations and decelerations (i.e., peak prominence >25 bpm) (Figure 2(b)). Irene's reactions were primarily reflected in changes in the rate of change of her peripheral skin temperature (Figure 2(c)).

**Figure 3:**
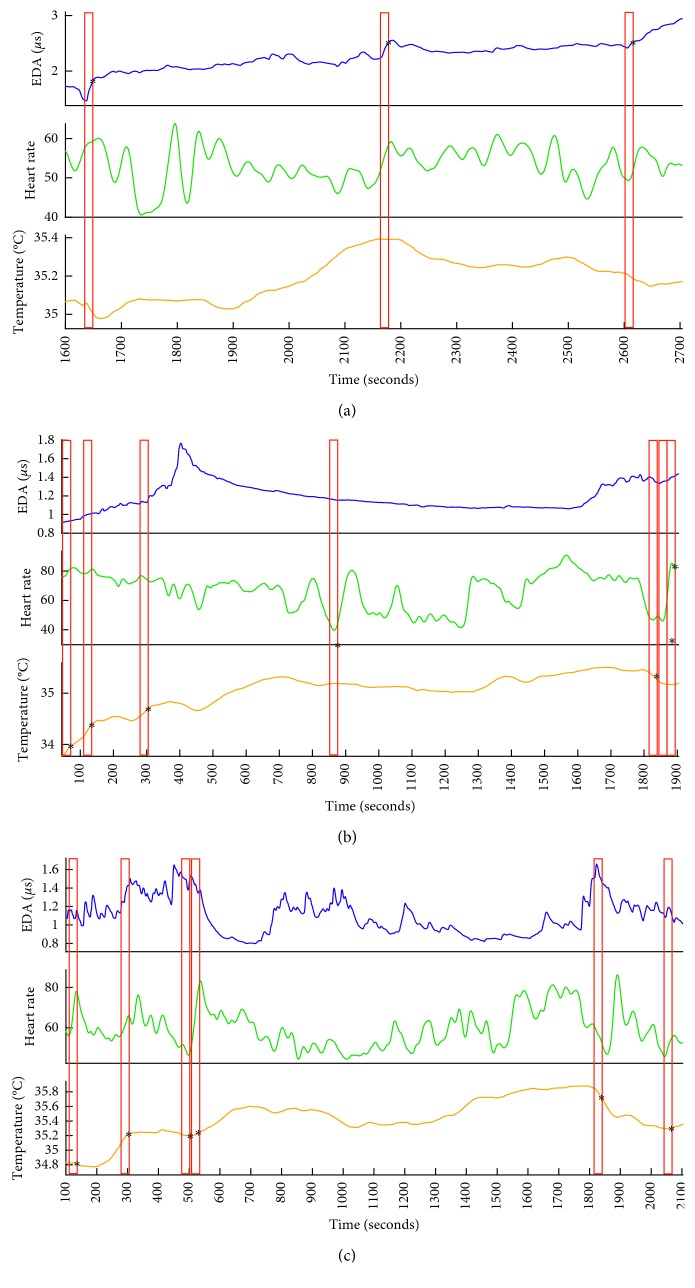
True-positive events detected for caregivers in their final movement session. Preprocessed and quality-checked electrodermal activity (blue), heart rate (green), and skin temperature (yellow) signals are presented for Liam (a), Giselle (b), and Sophie (c). Liam's significant events are triggered by electrodermal reactions; Sophie's by changes in vasodilatory and vasoconstriction responses in skin temperature. Giselle's significant events are triggered by a combination of both skin temperature and heart rate responses. The specific parameters for each individual's algorithm are presented in Supplementary Data, [Supplementary-material supplementary-material-1]. The unique patterns of responsiveness illustrate the need to tailor the event detection algorithm for caregivers.

Examples of the physiological responses that triggered the detection of an event for each person with dementia are presented in Figure 2 and for each caregiver in Figure 3.

### 3.2. Assistive Technology Can Identify Salient Events from Physiological Reactions

The performance of the assistive technology in identifying salient events was assessed through classifying each flagged event as either TP or FP, based upon video observation and participant feedback. Two research assistants independently classified each event; the interrater reliability was 96.6%.

**Figure 4:**
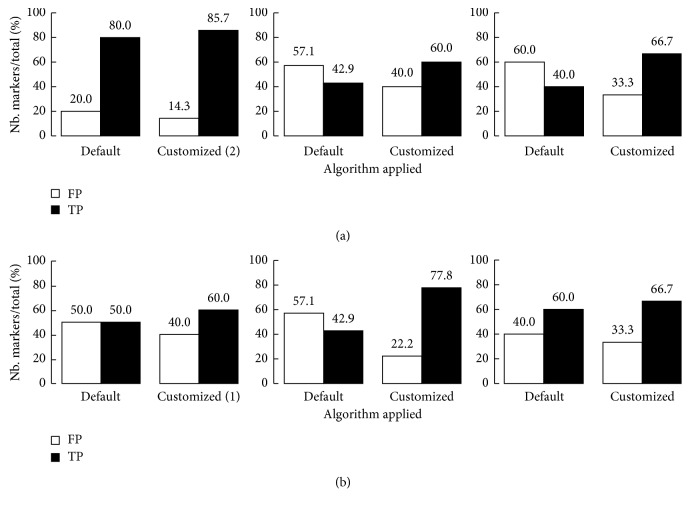
False-positive (FP) vs. true-positive (TP) markers identified by the default algorithm and the customized algorithm across chronological sessions. (a) A. Dyad 1 dementia participant Mary. B. Dyad 2 dementia participant Elisa. C. Dyad 3 dementia participant Irene. (b) A. Dyad 1 caregiver Liam. B. Dyad 2 caregiver Giselle. C. Dyad 3 caregiver Sophie.

Across all participants, 70% of significant moments detected by the best tailored *Event Finder* algorithm were true positives. The performance of the algorithm improved upon individual tailoring for both persons with dementia (Figure 4(a)) and their caregivers (Figure 4(b)).

### 3.3. Events Identified through Physiological Reactions Include Interpersonal Moments

**Figure 5:**
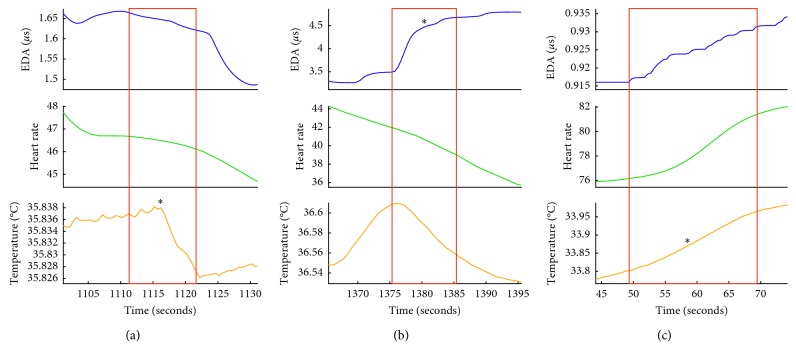
Examples of physiological signals associated with experiences of interpersonal significance. Red boxes highlight the detected event for (a) a moment of connection experience by an individual with dementia with her spouse; (b) a moment of recognition between an individual with dementia and a staff member; (c) a moment of connection experienced by a caregiver with her mother. ^*∗*^The physiological modality dominating the event-detection. However, the patterns of physiological changes triggering events differ within (a) and (b) and between (b) and (c) participants; all are associated with subjective experiences of interpersonal connection.

Within the TP events identified by *Event Finder*, TP_pers_ ranged from 40 to 89%. While the software predominant caught moments of TP_phys_ such as anxiety or pain over performing a specific movement, a significant portion was related to moments of interpersonal interaction. The physiological characteristics of three such events are presented in Figure 5, and the context of these events are described below. In contrast to Figure 4, where data were presented over the course of a long session (e.g., more than 10 minutes), Figure 5 illustrates a signal event detected in 30 seconds of physiological data.

The first event was triggered by a sharp decrease in temperature, identified by the algorithm as a negative 0.01°C change within 10 seconds (*T*_max_ = 35.8367°C and *T*_min_ = 35.8267; *t*_temp_ = 10) measured (Figure 5(a)). Prior to this moment, Mary was disengaged from the movement activity. Liam described the change in their interpersonal interaction that accompanied this event:

“I pretend to be pulled away from Mary. Then she gave me a light headbutt on my back. And that's when I touched her forehead with mine (forehead) back. She was playing!”

The second event was from dyad 1, triggered by Mary's EDR and shift from increasing to decreasing fingertip temperature (Figure 5(b)). Prior to this event, Mary had made eye contact with a staff member, Meredith, from the nonprofit organization, who she had known for several years but had recently forgotten. Liam describes the moment accompanying the physiological change:

“Oh, it was you! Meredith! You got a response. It was Meredith's engagement. Do you remember dear [to Mary] when Meredith was looking at you? You can see you and Meredith in the picture over there. And… when you saw her, you wanted to move with her.”

While Mary could not self-report about her experience of the moment, the significance of this event was described by several other participants. Meredith reported experiencing a significant connection with Mary during this moment:

“She looked at me and her face lightened up… something changed in her eyes and she smiled at me. I do not know how to say it… it's something you feel… I think it was recognition! Recognition! She recognized me… she remembered who I was.”

Sophie (caregiver in dyad 3) also reported that she noticed the significance of this moment. She began to cry as she explained what she witnessed: “I was so happy to see [Mary] happy, so that made me happy too. Because she was really smiling and she was really happy, and I thought, “Oh good for her!” Meredith got [Mary] up dancing… and [Mary] was enjoying it.”

The third event was from dyad 2, triggered by Giselle's increase of 0.17°C in skin temperature over 20 seconds (*T*_max_ = 33.96°C and *T*_min_ = 33.79; *t*_temp_ = 20) (Figure 5(c)). Giselle described the excitement that triggered this event: “I was watching [Elisa] move and realizing she's doing it more than usual. Sometimes she only gets into it later. I didn't realize she was still moving. I think she moved today more than she ever did!”

These examples demonstrate that the assistive technology is not only able to capture moments of physiological relevance but also able to capture moments of emotional and interpersonal significance.

## 4. Discussion

Intelligent assistive technologies (IATs) have emerged as promising tools to meet the escalating demands of caring for persons with dementia. While many wearable IATs have been developed for the purposes of tracking activity and maintaining functional and cognitive abilities, few technologies have focused on accessing the subjective experiences of individuals with dementia. In this study, we present a novel IAT that detects moments of significance for individuals with dementia through patterns of their physiological signals. This IAT is tailored to the individual's idiosyncratic response profile and is trained using the domain expertise of the caregiver with respect to the emotional state of the individual with dementia. We demonstrate that this wearable technology is able to accurately track significant moments associated with both physical and emotional events. Such a technology has the potential to accompany frameworks such as deep assessment [11] to improve care for people with advance dementia and to support the quality of life and care decisions of professional and more distal carers. Physiological markers of “significance” or meaning can support the understanding of what matters to persons with dementia, which is now core to health policy [28]. To accompany this article, we developed a freely available open-source toolbox for detecting significant events from physiological signals (http://www.github.com/BIAPT/Events-Finder). Our toolbox enables nonexperts to flag segments of a video recording with a high probability of physical or emotional salience, facilitating discussion and feedback of the event from the individual with dementia and their caregiver. The open-source nature of our toolbox allows researchers to customize individual functions for their needs and to incorporate individual sections into their respective analysis protocols.

Every individual has a distinct pattern of physiological responsiveness to salient stimuli. Thus, the performance of the IAT is dependent on the process of tailoring the parameters of the algorithm to an individual's idiosyncratic physiological characteristics. Tailoring the algorithm resulted in an increase in true-positive events and a decrease in false-positive events for both individuals with dementia and their caregivers (Figures 2(a) and 2(b)). The initial steps of this tailoring are time-consuming, as they involve reviewing video recordings synchronized with the physiological signals for visual moments of significance. However, this customizes the *Event Finder* algorithm so that it is sufficiently tuned to an individual's particular patterns of responsiveness to capture events with a high probability of salience (Figures 3 and 4). These events include both moments of physical significance and experiences of interpersonal significance (Figure 5). The latter steps of tailoring the algorithm parameters involved incorporating feedback from both the individual with dementia and their caregiver about the identified events. This combination of subjective self-report and third-person observation enabled us to incorporate the perspectives of individuals with dementia into the tailoring process and has been shown to be an effective strategy in the previous research with this population [26]. As symptoms of dementia include difficulties with abstract reasoning, such as remembering events and reflecting on their meaning [29], supporting the feedback process with video recordings and caregiver reflections was key to the latter stages of tailoring the algorithm parameters. Feedback from the participants was also key to identifying the valence of the event, which was less evident from the physiological data alone [30]. It was evident in the feedback process that caregivers were extremely attuned to the nonverbal behavior of the individual with dementia, which was an evocative means of self-communication and interpersonal communication [31]. Integrating their expertise into the behavior of the algorithm was a critical process in achieving high-performance accuracy in the detection of significant events. Furthermore, this integration may also facilitate the eventual acceptance of the IAT for everyday use, as low levels of user involvement in technology design is a codeterminant of low IAT adoption rates by individuals with dementia [3].

The IAT presented in this paper is one of a small handful IATs that use physiological signals to gather information about an individual with dementia. Smartwatches have been developed for this target population to use a combination of ANS and accelerometry signals to track physical activity [4, 32] as well as to detect changes in mental and emotional states. The technologies developed to-date use EDA alone [33] or in combination with heart rate, temperature and accelerometry signals to recognize stress and agitation in individuals with dementia [34, 35]. The IAT presented in this paper advances the capabilities of those developed to-date in two significant ways. First, existing IATs focus on tracking states of negative valence (e.g., stress and agitation) in individuals with dementia; we have demonstrated that our IAT can also detect moments of positive valence, including meaningful interpersonal connection, in individuals with dementia and their caregivers. Second, existing technologies focus on changes in overall state, such as activity and mood, and ignore transient, short-term changes. In contrast, our IAT is specifically tailored to detect “moments” of significance, which are transient by nature, in an effort to make visible what is meaningful to individuals with dementia [10]. By synchronizing the moments detected by physiological changes with 30-second video clips, our IAT enables individuals with dementia and their caregiver to focus on the transient events that are meaningful during an activity or an interaction, as opposed to their general baseline state. As such, our IAT advances the capabilities of existing technologies by highlighting events that signify what matters to an individual with dementia [11].

While the number of IATs designed for individuals with dementia double approximately every five years, less than 2% are intended to assist with the social and relational challenges associated with this condition [3]. Technologies that address interpersonal interactions are significantly more popular in other conditions, such as autism spectrum disorder [36, 37]. Moreover, IATs designed to detect emotions from physiological signals have been developed for decades in the field of affective computing [13, 38]. These technologies use advanced features calculated from ANS signals to train feature-based machine learning algorithms to detect discrete states of emotion [39, 40] or deploy an end-to-end deep learning approach for the dimensional recognition of emotions [41]. Drawing from the techniques and knowledge of these established fields will significantly accelerate the development of IATs that support the social and interrelation fabric of individuals with dementia.

This study has several limitations. First, the algorithm parameters were only tailored across the last four movement sessions. As some participants were absent during one or more sessions, this only allowed for two to three meaningful iterations of the algorithm parameters per participant. While each iteration improved Event Finder performance, we believe that the algorithm's performance would continue to improve with more sessions for tailoring the parameters and that the results reported herein do not represent the full capabilities of the Event Finder software. Second, the process we present for customizing the algorithm runs the risk of overfitting the parameter set to an individual's quotidian physiological characteristics. As the progression of dementia has been associated with changes in autonomic functions [42–44], it is possible that the algorithm would need to be retrained periodically on an individual with dementia as their physiological characteristics changed over time. Third, while signals from the autonomic nervous system are being recorded, it is important to not exclusively link the detected events with physiological responses. For example, clenching of the fists reflexively during significant moments has been associated with increases in electrodermal activity [16] and would have triggered an event detection by the *Event Finder* algorithm. Fourth, each physiological signal was processed independently, though it is known that interactions between the physiological modalities can discriminate between different mental and emotional states [45–47]. A multivariate approach to detecting significant changes in physiological state may result in better algorithm performance [48]. Finally, a limited number of features were extracted from each physiological modality; future improvements to the algorithm could involve the addition of features such as heart rate variability, which have shown strong correlation with an individual's emotional state [39].

## 5. Conclusions

We present a novel intelligent assistive technology (IAT) that detects salient physical and emotional events in individuals with dementia through patterns of their physiological signals. Our IAT consists of software that displays video recordings of events that are synchronized with potential moments of significance detected from physiological changes. Gathering feedback from individuals with dementia and their caregiver on their subjective experience of these moments enabled us to tailor algorithm parameters and improve algorithm performance. The algorithm was able to accurately detect moments of both emotional significance and moments of significant interpersonal connection in individuals with dementia. We provide our software as an open-source toolbox for the detection of significant physiological events from individually-tailored parameters in hopes that the assistive technology community will be able to benefit from and contribute to these tools.

## Figures and Tables

**Table 1:** Creating an artifact-detection algorithm to score the signal quality of physiological data.

ANS signal	Feature extracted	Threshold	SQI
Electrodermal activity	First derivative of signal over 15 s sliding window, incremented in 0.5 s intervals	Positive or negative change >3 *µ*s	0.4
	Flatness over 25 s sliding window, incremented in 0.5 s intervals	Difference between two consecutive points ≤0.001 *µ*s	0.1
	Out of normal physiological range	≤0.02 *µ*s	0
		>20 *µ*s	0.65
		>30 *µ*s	0

Skin temperature	Flatness over 25 s sliding window, incremented in 0.5 s intervals	Difference between two consecutive points ≤0.0001°C	0.5
	Out of normal physiological range	<15°C	0.5

**Table 2:** Participant description.

Participant with dementia	Caregiver	Dyad
Mary	Liam, spouse	1
Elisa	Giselle, daughter	2
Irene	Sophie, daughter	3

**Table 3:** List of sessions with marker feedback from dyad interviews.

Dyad	Session no.	Session ID	Max. no. of markers generated per individual
1	4	Default	5
	5	Customized (1)	5
	6	Customized (2)	7

2	6	Default	7
	8	Customized	10

3	4	Default	5
	8	Customized	10

**Table 4:** Creating customized algorithms for dementia participants from ANS signals.

ANS signal	Feature extracted	Thresholds	Scaling factor
*(A) Dyad 1 dementia participant Mary*			
Electrodermal activity	First derivative of signal over 10 s sliding window, incremented in 0.5 s intervals	Positive EDA change of 0.24 *µ*s	5
Heart rate	Local maxima and minima	Peak prominence of 20 bpm	0.05
Skin temperature	First derivative of signal over 15 s sliding window, incremented in 0.5 s intervals	Positive or negative temperature change of 0.05°C	1

*(B) Dyad 2 dementia participant Elisa*			
Electrodermal activity	First derivative of signal over 20 s sliding window, incremented in 0.5 s intervals	Positive EDA change of 0.25 *µ*s	4
Heart rate	Local maxima and minima	Peak prominence of 25 bpm	0.96
Skin temperature	First derivative of signal over 15 s sliding window, incremented in 0.5 s intervals	Positive or negative temperature change of 0.11°C	8.4

*(C) Dyad 3 dementia participant Irene*			
Electrodermal activity	First derivative of signal over 10 s sliding window, incremented in 0.5 s intervals	Positive EDA change of 0.25 *µ*s	4
Heart rate	Local maxima and minima	Peak prominence of 35 bpm	0.06
Skin temperature	First derivative of signal over 25 s sliding window, incremented in 0.5 s intervals	Positive or negative temperature change of 0.02°C	9

## Data Availability

The physiological data used to support the findings of this study are available from the corresponding author upon request.

## Supplementary Materials

Supplementary Materials Supplemental Table 1: creating customized algorithms for caregivers from ANS signals.
