# Supplementary material for: Wearable Technology for Detecting Significant Moments in Individuals with Dementia
Source: Biomed Res Int. 2019 Sep 25;2019:6515813. doi: 10.1155/2019/6515813 (PMC6778872; doi:10.1155/2019/6515813)
Supplement: Supplementary Materials — Supplemental Table 1: creating customized algorithms for caregivers from ANS signals. [file 6515813.f1.pdf]

SUPPLEMENTAL DATA

Supplemental Table 1: Creating customized algorithms for caregivers from ANS signals.

ANS signal	Feature extracted	Thresholds	Scaling factor
**A.**			
Electrodermal activity	First derivative of signal over 10 s sliding window, incremented in 0.5 s intervals	Positive EDA change of 0.2 µs	5
Heart rate	Peak and valley prominence	Positive or negative peaks at 12 bpm	0.15
Skin temperature	First derivative of signal over 10 s sliding window, incremented in 0.5 s intervals	Positive or negative temperature change of 0.05 °C	10
**B.**			
Electrodermal activity	First derivative of signal over 10 s sliding window, incremented in 0.5 s intervals	Positive EDA change of 0.45 µs	1.2
Heart rate	Peak and valley prominence	Positive or negative peaks at 25 bpm	0.1
Skin temperature	First derivative of signal over 20 s sliding window, incremented in 0.5 s intervals	Positive or negative temperature change of 0.12 °C	10
**C.**			
Electrodermal activity	First derivative of signal over 10 s sliding window, incremented in 0.5 s intervals	Positive EDA change of 0.25 µs	16
Heart rate	Peak and valley prominence	Positive or negative peaks at 20 bpm	0.06
Skin temperature	First derivative of signal over 20 s sliding window, incremented in 0.5 s intervals	Positive or negative temperature change of 0.037 °C	15

A. Dyad 1 caregiver, Liam. B. Dyad 2 caregiver, Giselle. C. Dyad 3 caregiver, Sophie.
